# SARS-CoV-2 antibody response after mRNA vaccination in healthcare workers with and without previous COVID-19, a follow-up study from the University Hospital in Krakow, Poland

**DOI:** 10.3389/fimmu.2022.1071204

**Published:** 2023-01-05

**Authors:** Izabella Owsianka, Agnieszka Pac, Estera Jachowicz, Karolina Gutkowska, Wiktor Szczuciński, Barbara Maziarz, Elżbieta Sochacka-Tatara, Piotr Heczko, Wojciech Sydor, Barbara Żółtowska, Jadwiga Wójkowska-Mach

**Affiliations:** ^1^ Doctoral School of Medical and Health Sciences, Jagiellonian University Medical College, Krakow, Poland; ^2^ Department of Microbiology, Faculty of Medicine, Jagiellonian University Medical College, Krakow, Poland; ^3^ Department of Epidemiology and Preventive Medicine, Faculty of Medicine, Jagiellonian University Medical College, Krakow, Poland; ^4^ Department of Diagnostics, Department of Clinical Biochemistry, Faculty of Medicine, Jagiellonian University Medical College, Krakow, Poland; ^5^ Center for Innovative Therapy, Clinical Research Coordination Center, University Hospital in Krakow, Krakow, Poland; ^6^ Department of Rheumatology and Immunology, Jagiellonian University Medical College, Krakow, Poland

**Keywords:** COVID-19, antibody levels, healthcare workers, COVID-19 immune response, mRNA vaccine against SARS-CoV-2

## Abstract

**Introduction:**

Healthcare workers (HCWs) from the beginning of the pandemic have been at risk of exposure to SARS-CoV-2, so they were vaccinated as first.

**Objectives:**

The purpose of the study was to determine the level of antibodies against SARS-CoV-2 in HCWs before and after vaccination with mRNA preparations according to previous COVID- 19.

**Patients and methods:**

The HCWs from the University Hospital in Krakow completed two surveys: the baseline survey before receiving the first dose of vaccine (in January 2021) and the follow-up survey in June 2021. In parallel, two blood samples were collected from each participant at baseline and at follow-up. Total anti-SARS-CoV-2 antibody levels were measured using the ECLIA technique.

**Results:**

At baseline, 41.1% of HCWs had positive antibody test results, and at follow-up, the vaccinated HCWs had almost 100 times higher antibody levels than the unvaccinated HCWs. Participants under 30 years of age had significantly higher antibody levels in June than older HCWs. Among participants with positive antibody test results in January, HCWs who had experienced asymptomatic COVID-19 had more than five times higher antibody levels in June than HCWs self-reported severe COVID-19. In total, 86.9% of HCWs received Comirnaty or Spikevax. The incidence rate of COVID-19 in the unvaccinated vs. vaccinated group was 13 times higher, 20.5% and 1.9% respectively.

**Conclusions:**

These results confirm the effectiveness of vaccination in the prevention of COVID-19 in HCWs. It is worth getting vaccinated regardless of previous infection. Furthermore, vaccination among HCWs under 30 years of age induced more effective antibody production compared to older individuals.

## Introduction

In the last few decades, we have witnessed the emergence of new infectious agents: coronaviruses (SARS-CoV, MERS-CoV), and quite recently the new SARS-CoV-2 coronavirus that causes disease in humans, COVID-19 (coronavirus disease 2019). According to WHO (World Health Organization) globally on 30 November, 640 395 651 confirmed cases of COVID-19 have been confirmed, including 6 618 579 deaths.

Fortunately, in a short time, specific vaccines have been developed and approved worldwide. As of 30 November 2022, a total of 13 042 112 489 vaccine doses have been administered (1). To date, more than 350 vaccine proposals for COVID-19 have been submitted, and more than 170 have entered clinical trials (2) but only seven of them have been approved for use in the European Union (Comirnaty - BioNTech/Pfizer, COVID-19 Vaccine Valneva- Valneva Austria GmbH Spikevax – Moderna, Vaxzevria – AstraZeneca, Jcovden - Janssen, Nuvaxovid – Novavax, VidPrevtyn Beta - Sanofi Pasteur) (3).

In Poland, according to WHO, from 3 January 2020 to 30 November 2022, there have been 6 353 850 confirmed cases of COVID-19 with 118 332 deaths. On 13 November 2022, a total of 57 715 479 vaccine doses were administered (4).

Health care workers (HCWs) are always at risk of acquiring infection from patients and subsequently exposing patients (5). The recent spread of COVID-19 globally led to considerable concern among healthcare workers about the dramatically increased probability of being infected when caring for infected patients. In fact, meta-analyses showed that a significant number of HCWs were reported to be infected with COVID-19 during the first 6 months of the COVID-19 pandemic, 15.1% of infected subjects required hospitalization and death occurs in 1.5% of infected patients (6, 7). The contact with infected individuals both inside and outside the hospital was associated with higher risk of SARS-CoV-2 infection among HCWs (8, 9) Fortunately, COVID-19 cases among health care workers decreased rapidly with the introduction of the anti- COVID-19 vaccination (10, 11).

In Poland, nationwide vaccinations began on 27 December 2020 from medical personnel. The Polish HCWs received one of the mRNA vaccines: Comirnaty or Spikevax or one of the two available recombinant vaccines: Vaxzevria or Jcovden.

The purpose of the study was to determine the level of antibodies against the SARS-CoV-2 spike antigen in the HCWs before and after vaccination with mRNA preparations depending on previous COVID-19. In addition, adverse reactions after vaccination were monitored.

## Patients and methods

### Study design

HCWs including nurses, paramedics, doctors, healthcare technicians, administrative staff and students at the University Hospital in Krakow, Poland, (UHK) were invited to participate in the study. Last-year students were only included in the study due to their participation in clinical rotations at the UHK during the pandemic and the completion of the entire pre-graduate medical education. HCWs were asked to complete two surveys: the first before receiving the first dose of vaccine or within 4 days thereafter (in January 2021) and the second survey after a 5-month follow-up (in June 2021). Two blood samples from each participant were collected, the first in January and the second in June. They were used to measure anti-SARS-CoV-2 antibodies. Altogether, 1484 HCWs were enrolled and written informed consents were obtained from all of them twice: January and June 2021. As many as 676 healthcare workers, who completed both surveys and whose blood samples were obtained twice, were included in the analysis. They were divided into two groups: vaccinated and unvaccinated. According to antibody levels in January, these two groups were split into four subgroups: (a) vaccinated with a positive antibody test (see below) result in January, (b) vaccinated with a negative antibody test result in January, (c) unvaccinated with a positive antibody test result in January and (d) unvaccinated with a negative antibody test result in January. Only participants who received two doses of the mRNA vaccines: Comirnaty or Spikevax (in January and February 2021) were analyzed in subgroups (a) and (b). Those vaccinated with Vaxzevria and Jcovden products were not included in a further analysis because they were given to only 22 HCWs and 3 HCWs, respectively. After merging the database comprising information from surveys with the database containing serological results, all data were pseudonymized. In December 2020, when the study was designed, only two doses were recommended by “European Medicine Agency’s (EMA) human medicines committee recommendations on vaccination against COVID–19”. This study was approved by the Bioethics Committee of Jagiellonian University protocol number 1072.6120.353.2020, date of approval 16.12.2020 and was conducted in accordance with the Declaration of Helsinki.

### Data collection, serologic testing, and data compilation

The baseline survey included questions on the sociodemographic characteristics of the participants e.g., the profession, the experience of work in healthcare in years. We also asked about the previous COVID-19 and the course of the infection. Participants could choose different symptoms which were stratified into 3 groups: asymptomatic infection (no symptoms); mild infection (fever, cough, fatigue, loss of smell or taste, body aches and pains, headache, sore throat, runny or stuffy nose, digestive symptoms including nausea, vomiting, or diarrhea) or severe (shortness of breath and difficulty breathing, pneumonia). The methods and population studied in the first stage of the study have previously been described in detail (8). In the follow-up survey, participants were also asked about vaccination, the type of COVID-19 vaccine and the number of doses received, and side effects after each dose. The side effects were classified as: local (eg. injections site pain, redness, or swelling), systemic (eg. fever, fatigue), serious (eg. anaphylaxis reaction) and listed in our survey in the multiple-choice question.

Venous blood samples were collected using a serum separator tube. The tubes were centrifuged at 4000 g for 15 minutes to separate the serum. Total Anti-SARS-CoV-2 S (Spike) antibody levels were measured using a commercially available fully automated Roche Cobas e801 immunoassay analyzer based on the electrochemiluminescence technique ‘ECLIA’ (Elecsys^®^ Anti-SARS-CoV-2 S, Roche, Mannheim, Germany, Cobas e analyzers) with the specificity and sensitivity 99.06% and 89.5%, respectively.

Positive test results at the beginning of January 2021 were defined as antibody values greater than or equal to 0.80 U/ml. Quantitative data interpretation was used to analyze the results of antibody tests in the follow-up in June 2021. Antibody levels above 25000 U/ml are qualified as equal to 25000 U/ml. The HCWs with a positive antibody test result in January were considered to have be infected with SARS-CoV-2 before enrollment in the study, regardless of whether they previously reported having COVID-19 in the initial survey. Asymptomatic infection was defined as the presence of antibody values greater or equal to 0.80 U/ml and the declaration of asymptomatic infection or lack of infection in the initial survey. The presence of antibody values greater or equal to 0.80 U/ml and the declaration of mild or severe symptoms of COVID-19 was described as symptomatic infection. Furthermore, the new SARS-CoV-2 infections occurring 14 days following the last dose reported in the questionnaire (in June) were the secondary outcome of this study.

### Statistical analysis

To describe the characteristics of the participants, the frequency with the corresponding percentages was used. The antibody concentration was presented as the geometric mean with geometric standard deviation due to a skewed distribution. McNemar’s test was used to compere the frequency of side effects after each dose. Mann-Whitney or Kruskal-Wallis (for variable with more than two categories) tests were used to investigate the relationship between antibody level after half-year of observation and potential factors related to them. *Post hoc* tests with Bonferroni correction were used for pairwise comparisons. The logistic regression model adjusted for age was used to assess the risk of COVID-19 based on anti-COVID-19 vaccination status and high levels of antibodies in January. A two-sided P-value of < 0.05 was considered statistically significant. All analyses were performed using IBM SPSS Statistics 28 (Chicago, US).

## Results

Together, 278 (41.1%) of the HCWs had a positive antibody test result in January 2021 during study enrollment. Among them, 167 people did not report COVID-19 before enrollment in the study and 15 reported that they had asymptomatic COVID-19, so in total 182 (65.5%) HCWs had asymptomatic infection. A total of 87 participants experienced mild symptoms during infection and 9 had severe disease. Furthermore, 87 participants reported previous infection, but had negative antibody test results, so they were included HCWs the group with a negative antibody test result in January.

In total, 588 participants (86.9% of the study population) received Comirnaty or Spikevax vaccines. The baseline characteristics of the population according to vaccination status are shown in **Table 1**. Together, 592 (87.6%) participants were women and 398 (59,0%) were over 40 years of age. Most of HCWs were nurses (52.7%), while medical doctors represented 5.9% and students 9.9%. A total of 280 (41.4%) participants had a master’s degree or equivalent and most of the study population (69.5%) worked in the healthcare system for over 5 years. As many as 278 (41.1%) of HCWs had a positive antibody test result in January 2021 (at the study beginning). Significantly more people received the vaccine against COVID-19 in the group with a negative antibody test result in January than in the group with a positive antibody test result in January: 90.2% vs 82.4%, respectively (p=0.003). However, no differences in vaccination status were observed between men and women or people of different ages, educational level, profession, and work experience in healthcare.

**Table 1:** Characteristics of the study population.

Descriptive Characteristics of Study Population	Total		Vaccination against COVID-19				p
			no		yes		
	N	%	N	%	N	%	
Sex							
women	592	87.6	77	13.0	515	87.0	0.982
men	84	12.4	11	13.1	73	86.9
Age N=675							
up to 30 years	173	25.6	15	8.7	158	91.3	0.159
31-40 years	104	15.4	20	19.2	84	80.8
41-50 years	188	27.9	24	12.8	164	87.2
51-60 years	171	25.3	24	14.0	147	86.0
>60 years	39	5.8	5	12.8	34	87.2
Educational level N=665							
At most high school	157	23.2	24	15.3	133	84.7	0.231
Post-high school	92	13.6	14	15.2	78	84.8
Bachelor	136	20.1	20	14.7	116	85.3
Master's or equivalent	280	41.4	27	9.6	253	90.4
Profession N=675							
Medical doctor	40	5.9	3	7.5	37	92.5	0.755
Nurse / midwife or paramedic	356	52.7	47	13.2	309	86.8
Administrative worker	108	16.0	13	12.0	95	88.0
Other	104	15.4	13	12.5	91	87.5
Student	67	9.9	11	16.4	56	83.6
Work experience in healthcare N=675							
< 12 months	56	8.3	7	12.5	49	87.5	0.924
1 - 5 years	150	22.2	18	12.0	132	88.0
> 5 years	469	69.5	62	13.2	407	86.8
positive antibody test result in January 2021							
no	398	58.9	39	9.8	359	90.2	0.003
yes	278	41.1	49	17.6	229	82.4

During follow-up, the odds of COVID-19 in the group of HCWs with negative antibody test results in January and not vaccinated (over the study period) were higher than among participants with negative antibody test result in January 2021 and vaccinated (OR 13.0; 95% CI = 4.41-38.17) (**Table 2**).

**Table 2:** Risk of self-reported COVID-19 according of vaccination status and previous COVID- 19 infection.

Serological status	COVID-19 after enrolment					
	no		yes		OR*	95% CI
	N	%	N	%		
antibodies –^1^ / vaccination -^3^	31	79.5	8	20.5	13.0	4.41-38.17
antibodies +^2^ / vaccination -^3^	47	95.9	2	4.1	2.1	0.43-10.61
antibodies – ^1^ / vaccination +^4^	352	98.1	7	1.9	1.0	ref.
antibodies +^2^ / vaccination +^4^	226	98.7	3	1.3	0.7	0.17-2.61

^1^negative antibody test result in January 2021.

^2^positive antibody test result in January 2021.

^3^not vaccinated in January 2021.

^4^vaccintaed in January 2021.

^*^age-adjusted odds ratio.

The antibody levels in June were measured on average 151.1 (+/- 8.1, min 23 max 176) days after the first dose of vaccine and 129.1 (+/-8.6, min 107 max 155) days after the second dose. Antibody levels in June 2021 did not differ between women and men. However, we have found a difference in antibody levels between different age groups at follow-up. HCWs, who were under 30 years of age, had significantly higher antibody levels in June than older HCWs in each age category: 31-40 years, 41-50 years, 51-60 years over 60 years. All vaccinated participants had antibody values greater than or equal to 0.80 U/ml in June.

Participants with positive antibody test result in January had a much higher antibody level in June than HCWs with negative antibody test result in January (1693.1 U/ml vs. 589.8 U/ml; p<0.001). Among the participants with a positive antibody test result in January, HCWs with asymptomatic infection had more than five times higher antibody levels in June than HCWs reported severe COVID-19 (1848.6 U/ml vs. 344.7 U/ml, p=0.046, **Table 3**).

**Table 3:** Antibody levels in June (U/mL) in relation to demographics factors, positive antibody test result in January 2021 and vaccination status.

	N	gMean	gSD	p_K-W_
Sex				
Women	592	921.1	7.97	0.775
men	84	835.6	9.55	
Age N=675				
up to 30 years	173	1330.3	6.75	<0.001
31-40 years	104	824.0	7.80	
41-50 years	188	748.6	8.70	
51-60 years	171	857.5	9.07	
>60 years	39	692.6	8.05	
Positive antibody test result in January 2021				
no	398	589.8	9.85	<0.001
yes	278	1693.1	4.96	
Course of COVID-19 N=278				
asymptomatic	182	1848.6	4.49	0.046
mild	87	1660.9	5.18	
severe	9	344.7	10.62	
Vaccination against COVID-19				
no	88	14.9	19.39	<0.001
yes	588	1684.6	2.50	
Side effects after vaccination N=588				
absent	97	1350.5	2.49	0.020
local	154	1563.9	2.47	
systemic	332	1857.3	2.49	
serious	5	1857.0	2.53	

gMean, geometric mean; gSD, geometric standard deviation.

Vaccinated HCWs had over 100 times higher antibody levels in June than unvaccinated HCWs 1684.6 U/ml vs. 14.9 U/ml (p<0.001). 332 participants experience systemic side effects, while 154 HCWs reported only local side effects (**Table 3**). Taking into account each symptom separately without considering other types of symptoms, the most frequent side effect after the first and second dose was injection site pain, 68.5% vs 53.1% respectively (p<0.001). Headache, as the most common systemic side effect, were more frequent after second dose (31.2%) vs. after the first dose (17.7%), p <0.001.

Furthermore, participants who experienced systemic side effects after vaccination had higher antibody levels in June than participants who did not report any side effects (1857.0U/ml vs. 1350.5 U/ml, respectively, p =0.02, **Table 3**). The difference between other groups according to type of side effects were not statistically significant.

**Figure 1:**
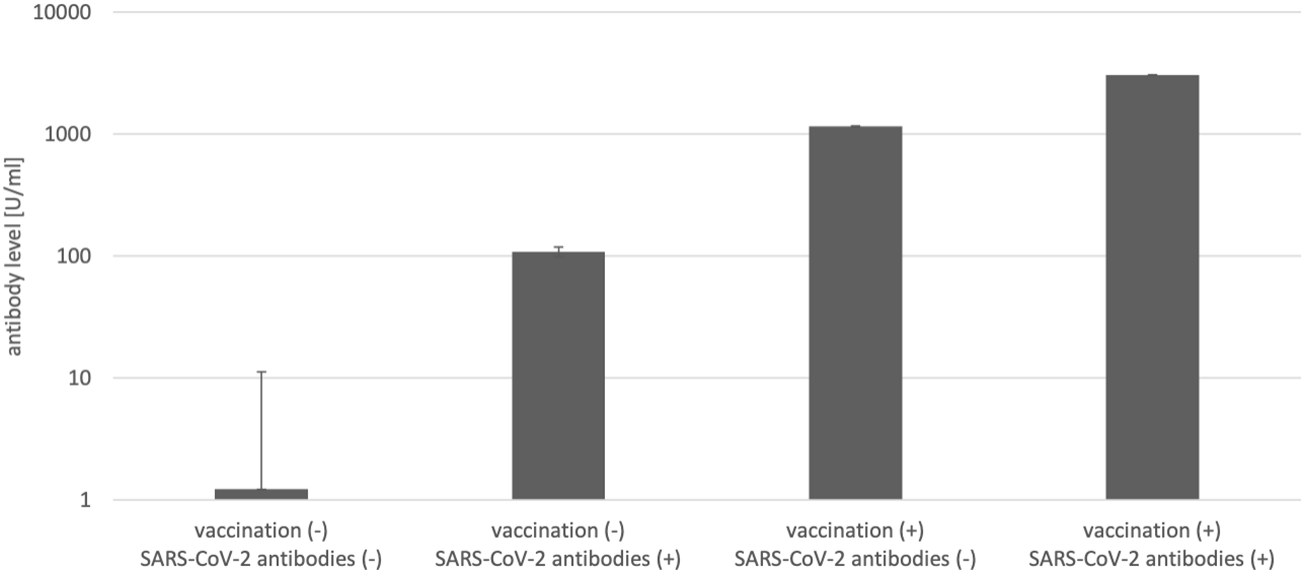
Antibody levels in June according to vaccination status and antibody test result in January 2021 (N=656).

Antibody levels in June according to vaccination status and positive antibody test result in January are shown in **Figure 1**. The HCWs who were infected with SARS-CoV-2 after enrollment were excluded from this analysis. The vaccinated HCWs with the negative test result in January had over ten times higher antibody levels in June than unvaccinated HCWs with positive antibody test results in January 2021. Vaccinated participants with a positive antibody test result at baseline had almost 30 times higher antibody levels in June than unvaccinated participants with a positive antibody test result at the start point.

## Discussion

The June follow-up survey revealed that 86.9% of participants in our Polish study were vaccinated with two doses of Pfizer-BioNTech or Spikevax vaccines. EMA recommendations on extra doses and boosters were published 4 October 2021, thus the analysis of antibody levels after booster doses was not included in the study (12). Based on data acquired from the European Center for Disease Prevention and Control website, regional public health agencies, and other studies, this rate was similar to the median value – 88% - of the vaccination rates of EU/EEA countries for fully vaccinated (13, 14). Compared to individual countries, the HCWs vaccination rates in our study were lower than those of Canada – 92% (13), and higher than those reported from France – 71% (15), United Kingdom – 75% (14) and United States – 70% (16) It should be noted that up until March 1, 2022, vaccinations were not mandatory for HCWs in Poland. These results show that vaccine hesitancy was not widespread among HCWs in this particular hospital, unlike among other Poles – in December 2022 less than 60% of the Polish population were fully vaccinated (17, 18). It is of interest that the acceptance rate among our HCWs was even higher than reported in a multicenter study conducted in Canada (19). According to Luo et al. (20) male HCWs, 30 years or older, were more likely to be vaccinated against COVID-19. However, in our study, there were no differences in vaccination status between men and women or people of different ages and educational levels. One possible contributor to this finding is that the HCWs in Poland were afraid that vaccine supply might be limited, so they did not hesitate to get vaccinated.

In our study, up to 41.1% of HCWs had positive antibody test results, before the vaccinations started which may suggest that their exposure to the virus was indeed very high, as it was previously described (8). According to Afzal et al. only 0.028% results of ECLA test can be false positives (21). Somewhat, the obtained results may be explained by the potentially greater intensity of the contact between HCWs and patients in Polish hospitals than in other countries (8). Quite recently, our colleagues from the Polish National Institute of Public Health showed in their multicenter study that the seroprevalence for Polish HCWs working in hospitals but also in ambulatory practices before the vaccination campaign was 25.2% (22). Moreover, they revealed that reporting a household contact and contact in a workplace, including a contact with a positive patient predicted significantly the positive result of antibody test (22). In Poland, the number of nurses, who represented most of the individuals in our study, per 100 hospital beds is five times less than in other European countries (23), therefore they spent with patients more time.

Fortunately, the immunological response to vaccination in our HCWs in all subgroups was very good, as antibody titers reached levels of over 2000 U/ml. These values correspond to those obtained by Uysal et al. in their studies on a similar HCWs group in a single hospital in Turkey, although they were vaccinated with inactivated virus vaccine (24). These differences raise the question of possible immunity waning after vaccinations in HCWs using different vaccines and also of the relevance between antibody levels to spike protein and protection against Covid-19. Generally, antibodies against SARS-CoV-2 in HCWs after vaccination have been shown to last at least 6–8 months (25).

Obviously, vaccination played a key role in protecting HCWs against Covid-19, because, as our study showed, it reduced the risk of SARS-CoV-2 infection. In addition, similar results were obtained by other groups (5, 26). On the other hand, comparative studies on HCWs in Hong Kong immunized with mRNA Comirnaty versus inactivated Coronavac vaccines showed, using both ELISA anti-spike protein and neutralizing antibody assays, that the mRNA vaccine caused substantial increase in antibody levels even after the first dose on compared to the inactivated vaccine, which caused low antibody response (26).

Vaccination induces antibody production, which represents the humoral immune response. In our study, individuals with a previous SARS-CoV-2 infection had higher antibody levels 6 months after vaccination than HCWs without contact with SARS-Cov-2. Similar results were observed by Milazzo et al. In their study anti-SARS-CoV-2 spike antibody levels after one and two doses of Comirnaty were statistically significant higher in SARS-CoV-2-experienced than in SARS-CoV-2-naïve HCWs (27).

On the other hand, there were our hospital staff members who got the infection before the vaccinations started in the hospital and then they were twice vaccinated. They may represent the subpopulation with the highest protection which may be not fully related to the antibody titers. It is known that even two doses of the mRNA vaccine are associated with high short-term protection against SARS-CoV-2 infection; this protection wanes after 6 months while infection-acquired immunity boosted with vaccination may remain high more than 1 year after infection (28, 29). However, according to DiPiazza et al., protective immunity against Covid-19 based on T cells activation may be effective in individuals who do not show very high antibody titers in standard assays (30)

Although, to date, there is no multicenter study-based threshold specifying which titers ensure protection against COVID-19, Feng et al. showed that higher levels of binding and neutralizing antibodies are associated with a lower risk of symptomatic disease (31). Furthermore, Goldblatt et al. found that the mean protective threshold of the IgG anti-Spike antibody concentration for the wild-type virus was 154 Binding Antibody Units (BAU/ml) assessed by ELISA (32). More studies are needed in this field to draw clear conclusions.

The antibody response varied among different age groups. Participants under 30 years of age had significantly higher antibody levels in June than older HCWs. Similar, Ammar et al. observed that HCWs under 36 years of age had antibody titer levels higher than those of older participants (33). The differences are likely a consequence of immunosenescence, age-related involvement of the immune system, leading to a progressive reduction in the ability to trigger effective antibody production against, e.g., vaccinations (34). Similarly, a limited response to vaccination in elderly individuals was also observed in case of immunization against influenza (35, 36).

It should be stressed also that medical students of the last year who thanks to clinical rotations were included to the specific university hospital environment. Although vaccine hesitancy among them was not a recorded for the purpose of this study, they were uniformly enthusiastic as their American colleagues as reported recently (37). Moreover, like students of the Birmingham Medical School (38) our medical students got the learning opportunities in clinical research when they helped us in this study. As the result of our experience gathered during this study, we are organizing an education program that integrates medical and nursing students into interdisciplinary teams to train them in infection control with special regards to vaccinations for HCW and patients.

### Strength and limitations

A key strength of the present study was the large population of medical staff and last-year medical students. In addition, all participants were vaccinated in a short period of time.

However, this research also has some limitations. It is a single-center study in which we only analyze the impact of mRNA vaccines. In December 2020, when the study was designed, only two doses were recommended by “European Medicine Agency’s (EMA) human medicines committee recommendations on vaccination against COVID–19”. EMA recommendations on extra doses and boosters were published 4 October 2021, thus the analysis of antibody levels after booster doses was not included in the study. Secondly, the follow-up was short and the findings concerning the effectiveness of vaccination in COVID-19 prevention may not apply to infections with new variants of SARS-CoV-2, eg, Omicron. Finally, in the analysis of the course of COVID-19 we based on the statements of the participants in the baseline survey without clinical confirmation.

## Conclusions

These results confirm the effectiveness of vaccination in the prevention of COVID-19 in HCWs. It is worth getting vaccinated even after infection with SARS-CoV-2. Furthermore, vaccination among workers under 30 years of age induced more effective antibody production compared to older individuals. It should be of importance to perform follow-up studies in the next periods of time along with the next revaccinations on the same HCWs population to obtain valuable data on the persistence of the antibody response and its relevance to protect such an exposed group from possible next waves of Covid-19.

## Data availability statement

The raw data supporting the conclusions of this article will be made available by the authors, without undue reservation.

## Ethics statement

The studies involving human participants were reviewed and approved by the Bioethics Committee of the Jagiellonian University (protocol code 1072.6120.353.2020, date of approval 16.12.2020). The patients/participants provided their written informed consent to participate in this study.

## Author contributions

Conceptualization, IO and JW-M; Methodology, WS, EJ, and JW-M; Software, AP and ES-T; Formal Analysis, IO, AP, ES-T, and JW-M; Investigation, BZ, WS, BM, and JW-M; Resources BZ, and JW-M; Data Curation, IO, WSz, KG, and JW-M; Writing Original Draft Preparation: IO, WSz, PH, and JW-M; Writing, Review and Editing: IO, PH, and JW-M; Supervision, JW-M; Project Administration, WS and JW-M; Review and revision of the manuscript: PH and JW-M; Acquisition of funds for publication: WS and JW-M. All authors have read and agreed to the published version of the manuscript.
