# Corrigendum: SARS-CoV-2 antibody response after mRNA vaccination in healthcare workers with and without previous COVID-19, a follow-up study from the University Hospital in Krakow, Poland

**DOI:** 10.3389/fimmu.2023.1155871

**Published:** 2023-03-07

**Authors:** Izabella Owsianka, Agnieszka Pac, Estera Jachowicz, Karolina Gutkowska, Wiktor Szczuciński, Barbara Maziarz, Elżbieta Sochacka-Tatara, Piotr Heczko, Wojciech Sydor, Barbara Żółtowska, Jadwiga Wójkowska-Mach

**Affiliations:** ^1^Doctoral School of Medical and Health Sciences, Jagiellonian University Medical College, Krakow, Poland; ^2^Department of Microbiology, Faculty of Medicine, Jagiellonian University Medical College, Krakow, Poland; ^3^Department of Epidemiology and Preventive Medicine, Faculty of Medicine, Jagiellonian University Medical College, Krakow, Poland; ^4^Department of Diagnostics, Department of Clinical Biochemistry, Faculty of Medicine, Jagiellonian University Medical College, Krakow, Poland; ^5^Center for Innovative Therapy, Clinical Research Coordination Center, University Hospital in Krakow, Krakow, Poland; ^6^Department of Rheumatology and Immunology, Jagiellonian University Medical College, Krakow, Poland

**Keywords:** COVID-19, antibody levels, healthcare workers, COVID-19 immune response, mRNA vaccine against SARS-CoV-2

In the published article, there was an error in the article title. Instead of “SARS-CoV-2 antibody response after mRNA vaccination in healthcare workers with and without previous COVID-19, a follow-up study from a university hospital in Poland during 6 months 2021”, it should be “SARS-CoV-2 antibody response after mRNA vaccination in healthcare workers with and without previous COVID-19, a follow-up study from the University Hospital in Krakow, Poland”.

The authors apologize for this error and state that this does not change the scientific conclusions of the article in any way. The original article has been updated.

